# Correlation between DNA ploidy, metaphase high-resolution comparative genomic hybridization results and clinical outcome of synovial sarcoma

**DOI:** 10.1186/1746-1596-6-107

**Published:** 2011-11-03

**Authors:** Zsófia Balogh, Zsuzsanna Szemlaky, Miklós Szendrői, Imre Antal, Zsuzsanna Pápai, László Fónyad, Gergő Papp, Yi C Changchien, Zoltán Sápi

**Affiliations:** 1 1st Department of Pathology and Experimental Cancer Research, Semmelweis University, Budapest, Hungary; 2 Semmelweis University Orthopedic Clinic, Budapest, Hungary; 3 Military Hospital-State Health Centre Department of Oncology, Budapest, Hungary

**Keywords:** High-Resolution Comparative Genomic Hybridization, HR-CGH, synovial sarcoma, DNA ploidy, clinical outcome, SYT, SSX

## Abstract

**Background:**

Although synovial sarcoma is the 3rd most commonly occurring mesenchymal tumor in young adults, usually with a highly aggressive clinical course; remarkable differences can be seen regarding the clinical outcome. According to comparative genomic hybridization (CGH) data published in the literature, the simple and complex karyotypes show a correlation between the prognosis and clinical outcome. In addition, the connection between DNA ploidy and clinical course is controversial. The aim of this study was using a fine-tuning interpretation of our DNA ploidy results and to compare these with metaphase high-resolution CGH (HR-CGH) results.

**Methods:**

DNA ploidy was determined on Feulgen-stained smears in 56 synovial sarcoma cases by image cytometry; follow up was available in 46 cases (average: 78 months). In 9 cases HR-CGH analysis was also available.

**Results:**

10 cases were found DNA-aneuploid, 46 were DNA-diploid by image cytometry. With fine-tuning of the diploid cases according to the 5c exceeding events (single cell aneuploidy), 33 cases were so called "simple-diploid" (without 5c exceeding events) and 13 cases were "complex-diploid"; containing 5c exceeding events (any number). Aneuploid tumors contained large numbers of genetic alterations with the sum gain of at least 2 chromosomes (A-, B- or C-group) detected by HR-CGH. In the "simple-diploid" cases no or few genetic alterations could be detected, whereas the "complex-diploid" samples numerous aberrations (equal or more than 3) could be found.

**Conclusions:**

Our results show a correlation between the DNA-ploidy, a fine-tuned DNA-ploidy and the HR-CGH results. Furthermore, we found significant correlation between the different ploidy groups and the clinical outcome (p < 0.05).

## Background

Synovial sarcoma (SS) is the 3rd most common mesenchymal spindle cell tumor [1]. It occurs most commonly in the young, representing about 8% of all soft tissue sarcomas but about 15-20% of cases in adolescents and young adults [2]. The peak of incidence is before the 4th decade and males are affected more often than females (ratio around 1.2:1). Almost all SSs are high-grade lesions with a 5-year overall survival rate of 36-76%, depending on the patient's age, the tumor size, the proportion of poorly differentiated areas and the resectability of the tumor. Histologically, two distinct subtypes can be distinguished: a monophasic, containing spindle cells; and a biphasic, containing both an epithelial and a spindle cell component [3]. A rare form of monophasic SS also exists, containing only epithelial-like cells [4]. The monophasic subtype is more common than the biphasic one. SS has a characteristic balanced translocation between chromosomes × and 18, t(X;18) (p11.2;q11.2), represented in more than 95% of the cases. The translocation fuses the *SYT *gene from chromosome 18 to either of three highly homologous genes at Xp11: *SSX1, SSX2 *or *SSSX4. SYT-SSX1, SYT-SSX2 *and *SYT-SSX4 *are thought to function as transcriptional regulators [5]. It has been shown that the type of fusion transcript most probably correlates with the clinical outcome and *HER-2 *oncogene amplification is associated with a lower risk of developing metastasis [6-8]. According to the comparative genomic hybridization (CGH) data published in the literature, simple and complex karyotypes show correlation with the prognosis [9,10], whereas the connection between DNA ploidy and clinical course is controversial [11]. For this reason, we performed a fine-tuned interpretation of our DNA ploidy results and compared these to high-resolution comparative genomic hybridization (HR-CGH) outcome, by using the same samples. We also examined, whether there is an association between the ploidy status and the metastasis or recurrence of the tumor.

## Methods

### Tumor samples

56 synovial sarcoma cases were selected for this study. All cases were classified according to the World Health Organization (WHO) classification of tumors of soft tissue [1]. In the specimens selected for CGH analysis, the proportion of tumor cells were estimated after hematoxylin-eosin staining of tissue sections preceding to DNA extraction. In all cases the tumor cells comprised at least 85% of the total tissue areas. One part of the surgically resected tumor tissues were frozen at -80°C. The other parts of the tumors were in formalin-fixed and paraffin-embedded status. Furthermore, in some cases fresh-made smears were available. Follow up was available in 46 cases (average: 78 months) with an interval of 38 to 92 months. The clinical data of patients are summarized in Table 1. All patients' information was coded, with complete clinical data available only for physicians involved in the treatment of these patients. This study was approved by the ethics committee of the Semmelweis University (approval number: 230-151/2006-1018EKU), and all patients gave their informed consent.

**Table 1:** Clinical data and result of DNA content analysis of 56 patients with synovial sarcoma

**No**.	Sex	Age (Years)	Diagnosis	DI	Histogram	Follow-up
1	M	41	MSS	1.15	A	M
2	M	33	PSS	1.21	A	M(D)
3	F	63	MSS	1.08	Dc	M
4	F	48	MSS	0.96	Dc	N
5	F	38	BSS	0.96	Dc	R
6	F	28	BSS	0.93	Ds	N
7	M	13	MSS	1.07	Ds	N
8	M	25	MSS	0.95	Ds	M
9	M	55	PSS	1	Ds	N

10	F	22	MSS	0.95	Ds	M
11	F	47	MSS	0.91	Ds	M
12	M	22	MSS	1.01	Ds	M(D)
13	M	39	MSS	1.06	Ds	M(D)
14	M	67	BSS	0.96	Ds	R
15	F	52	MSS	0.92	Ds	M
16	M	31	MSS	0.93	Ds	N
17	F	11	BSS	0.97	Ds	M(D)
18	F	41	MSS	1	Ds	R
19	F	42	MSS	0.98	Ds	M(D)
20	M	48	MSS	0.96	Ds	M
21	F	62	MSS	0.93	Ds	M
22	F	20	MSS	1.05	Ds	M
23	M	22	MSS	1.04	Ds	N
24	M	56	BSS	0.98	Ds	R
25	F	13	BSS	0.95	Ds	N
26	M	43	MSS/PSS	0.94	Ds	R
27	F	58	MSS	0.9	Ds	M
28	M	36	MSS	1.09	Ds	R
29	M	41	MSS	0.92	Ds	R
30	M	43	MSS	0.97	Ds	R
31	F	32	BSS	0.99	Ds	N
32	F	68	MSS	0.99	Ds	N
33	F	68	MSS	1.64	A	M
34	F	29	MSS	1.85	A	M
35	F	21	PSS	1.17	A	M
36	F	58	PSS	1.51	A	M
37	M	37	MSS/PSS	1.6	A	M
38	M	60	MSS	1.26	A	M
39	M	58	PSS	1.34	A	M
40	F	31	MSS	1.19	A	M(D)
41	M	42	MSS	1	Dc	M(D)
42	F	29	MSS	1.08	Dc	M
43	M	37	PSS	1.07	Dc	M
44	M	46	MSS	1.04	Dc	N
45	M	34	PSS	1.03	Dc	M
46	F	32	MSS	0.95	Dc	R

47	M	23	BSS	0.95	Ds	ND
48	M	52	MSS	1.02	Ds	ND
49	M	14	BSS	0.95	Ds	ND
50	M	42	PSS	0.92	Ds	ND
51	F	56	MSS	1.03	Ds	ND
52	F	25	BSS	1.02	Ds	ND
53	M	32	MSS	0.95	Dc	ND
54	F	23	PSS	0.94	Dc	ND
55	M	24	BSS	1.03	Dc	ND
56	F	70	PSS	1.06	Dc	ND

Abbreviations: DI, DNA index; MSS, monophasic synovial sarcoma; BSS, biphasic synovial sarcoma; PSS, poorly differentiated synovial sarcoma; M, metastasis; R, only recurrence; M(D), died because of tumor; N, no recurrence and no metastasis; ND, no data; A, aneuploid; Ds, diploid simple (diploid stemline without 5c exceeding events); Dc, diploid complex (diploid stemline with any cell of 5c exceeding events)

### DNA smear image cytometry/DNA image analysis

The DNA smear image cytometry was performed on 56 synovial sarcoma cases. The fresh-made smears were ready to use, the deep-frozen and the formalin-fixed and paraffin-embedded tissue samples needed preceding preparations. Single-cell layer smears were prepared on glass slides from the frozen tissue samples. The fresh-made and single-cell layer smears were post-fixed in 4% buffered formalin for 30 min. From the formalin-fixed and paraffin-embedded samples the nuclei were isolated before the smears were prepared. Three 50 μm sections were cut from each paraffin block. The sections were deparaffinized in xylene and rehydrated in a series of decreasing concentration of ethanol. The tissue was digested in Carlsberg's solution (0, 1% Sigma protease XXIV; 0, 1 M Tris buffer; 0, 07M NaCl; pH 7, 2) for 45 min at 37°C in thermomixer. After filtration the nuclear extract was washed in distilled water, the nuclei were fixed in 70% ethanol and dropped on glass slide to prepare smear.

The nuclear DNA content was analyzed by DNA image cytometry, based on adsorption technique. In order to make the nuclear extract suitable for quantitative analysis we stained the samples by a stoichiometric method according to Feulgen using Schiff reagent (Merck, Darmstadt, Germany). Sample preparation, fixation and staining were performed according to the consensus report of the ESAP task force on the standardization of diagnostic DNA image cytometry [12]. DNA image data processing was carried out using a regular microscope with an image-sensing scanner, interfaced to a regular PC with dedicated software installed (CYDOK R, Fa., Hilgers, Königswinter, Germany). We used a 40x objective and an interference filter (565 ± 10nm). At least 200 diagnostic cells were analyzed on each smears. The integrated optical density (IOD) of Feulgen-stained reference cells (e.g. lymphocytes or granulocytes) were used as an internal standard for the normal diploid DNA content (2c) to rescale the IOD values into c-values. The coefficient of variation of the reference cells was between 3% and 5%. The reference cells were non tumor cells found in the samples. Having measured 30 reference cells and 200 tumor cells, image analysis histograms were generated. A DNA index (DI) 1 corresponds to the 2c diploid DNA content and the DI of 2.5 to the 5c DNA content. Between DI of 0.9-1.1 the sample is considered to be diploid. Outside of this range the tumor is considered to be aneuploid. To define DNA-aneuploidy we used the stem line interpretation of Haroske *et al*. [12]. Single cell aneuploidy (complex diploid tumor) was defined as the tumor had a diploid stemline but we found any cell among 5c exceeding events - excluding eusomic polysomy (8c +/- 0.4c) - the case was considered as complex diploid. [13]

### Metaphase high-resolution comparative genomic hybridization (HR-CGH)

All of the 9 cases of synovial sarcoma, from which fresh frozen tissue samples were available, HR-CGH analysis was performed. DNA was extracted with a standard salting out method according to Miller *et al*. [14]. Hybridization was performed according to a standard procedure [15]. Briefly, sex-matched normal and tumor DNAs were labeled with SpectrumRed-dUTP and SpectrumGreen-dUTP, respectively by nick translation using a commercial kit (Vysis, Downers Grove, IL, USA). Hybridization was performed in a moist chamber at 37°C for 3 days in the presence of an access of blocking Human Cot-1 DNA (Vysis). The metaphases were captured with epifluorescence microscope (Nikon Eclipse E600) equipped with a monochrome CCD camera, and were analyzed using LUCIA CGH-Advanced Statistics 1.5.0 software (Laboratory Imaging Ltd, Prague, Czech Republic). At least 15 karyotypes were analyzed in each case. Ratio profiles were evaluated by dynamic Standard Reference Interval based on an average of 15 normal CGH analyses using 16 normal DNAs instead of conventionally fixed thresholds. The gains and losses were detected by comparing the 99.5% confidence interval of the mean ratio profiles of the test samples to the 99.5% dynamic standard reference interval [16]. We have applied the correction for unreliable hybridizations function to improve the specificity of genomic gains and losses detection. Negative and positive controls were included in each experiment. Two differentially labeled DNAs (normal test and normal reference) hybridized together served as a negative control. Positive control was created from the specimens with a known trisomy 21, and partial deletion of chromosome 17, del(17)(p13). Heterochromatic regions in the centromeric and paracentromeric areas of the chromosomes, the short arm of the acrocentric chromosomes, and the Y heterochromatic as well as telomeric regions were not included in the evaluation.

### Statistics

For statistical analysis the Fisher's exact test was used. Differences were considered statistically significant when P value was < 0.05.

## Results

**Figure 1:**
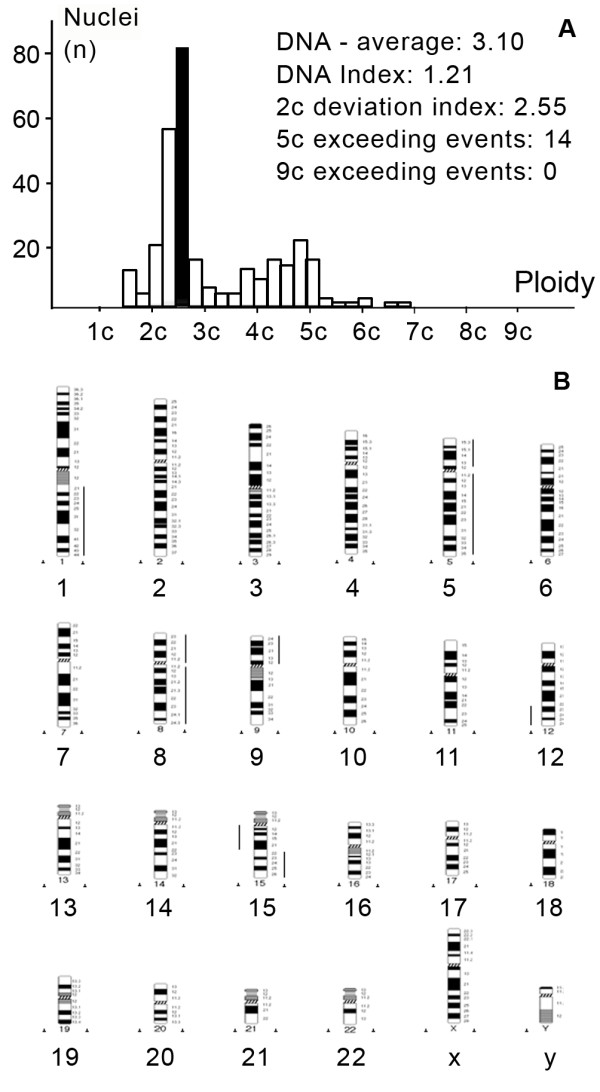
**DNA histogram (a) and HR-CGH ideogram (b) of aneuploid synovial sarcoma (case 2)**. Aneuploid tumor with a complex gain (1q, +5, +8, 9p, 12p13-q22, 15q22-26) and loss (12q23-24, 15q11.2-21) of DNA content. DNA index was found to be 1.21. The lines at the right of the chromosome ideogram represent DNA gains, lines at the left, DNA losses.

**Figure 2:**
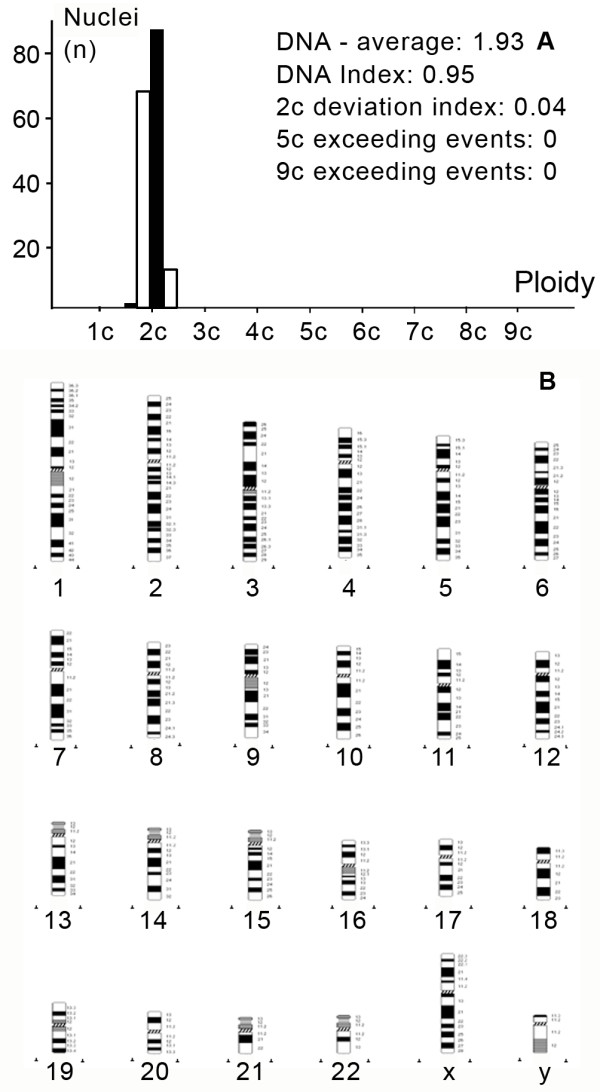
**DNA histogram (a) and HR-CGH ideogram (b) of a simple diploid synovial sarcoma (case 8)**. Note there is no cell above the 5c value and there is no alteration concerning the CGH results.

**Figure 3:**
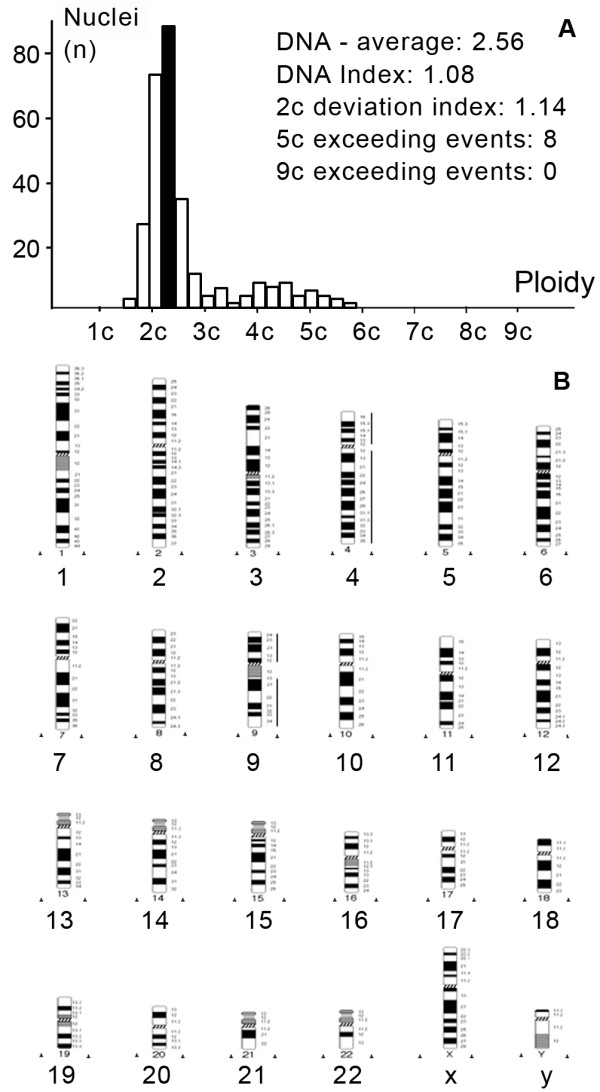
**DNA histogram (a) and HR-CGH ideogram (b) of a complex diploid synovial sarcoma (case 3)**. Though the tumor is diploid, 8 cells proved to be above the 5c value. CGH revealed the gain of the whole chromosomes of 4 and 9. The lines at the right of the chromosome ideogram represent DNA gains.

10 cases (17.9%) were aneuploid by image cytometry, the average DI was 1.40 (Figure 1). The histogram of 46 neoplasms (82.1%) represented diploid tumor with 0.99 mean DI. Fine-tuned analysis was performed on diploid cases according to the 5c exceeding events which represent single cell aneuploidy. 33 cases (71.7% of diploid cases) without 5c exceeding events were considered "simple-diploid" (Figure 2) and 13 cases (28.3% of diploid cases) fell into the "complex-diploid" group containing any 5c exceeding events (Figure 3). These results are shown in Table 1. Regarding the cases having been followed-up we had 10 aneuploid, 9 complex-diploid and 27 simple-diploid synovial sarcomas.

Only aberrations that are present in a high proportion of tumor cells can be detected by CGH. If the normal cell content is more than 50% of the tumor specimen, the evaluation becomes difficult. The average tumor cell concentration in this material was 85%. This evaluation and selection of representative tumor material is extremely important for the outcome of CGH.

HR-CGH analysis revealed chromosome imbalances in 5 out of the 9 (55.5%) SSs; in 4 cases neither chromosomal gains nor losses were found. The chromosomal imbalances found are listed in Table 2. Aneuploid tumors contained a large number of genetic alterations with the sum gain of at least 2 chromosomes (A-, B- or C-group). These single alterations consisted of gains of chromosome were 1p, 1q, 2q24-q37, 5, 7q11.2-q36, 8, 8q23-24, 9p, 12p13-q22 and 15q22-26 and losses of chromosome were 3p14-p26, 4, 5p14, 12q23-24, 13q12-q22, 15q11.2-21, 16q and 18q12-q22 (Figure 1). The "complex-diploid" samples showed numerous aberrations (equal or more than 3) such as gains of chromosome 3q, 4 and 9, and losses of chromosome 1p12-22, 3p, 4q28-q35, 5q15-35, 6q12-q23, 16q, 19, 20 and 22 (Figure 3). In "simple-diploid" cases no genetic alterations could be detected by HR-CGH (Figure 2).

**Table 2:** High-resolution comparative genomic hybridization(HR-CGH) data of 9 synovial sarcoma patients

**No**.	Histogram	Gain	Loss
1	A	1p, 2q24-q37, 7q11.2-q36, +8, 8q23-24	3p14-p26, -4, 5p14, 13q12-q22, 16q, 18q12-q22
2	A	1q, +5, +8, 9p, 12p13-q22, 15q22-26	12q23-24, 15q11.2-21
3	Dc	+4, +9	-
4	Dc	3q	1p12-22, 3p, 5q15-35, -19, -20, -22
5	Dc	-	4q28-q35, 6q12-q23, 16q
6	Ds	-	-
7	Ds	-	-
8	Ds	-	-
9	Ds	-	-

Abbreviations: A, aneuploid; Dc, diploid-complex; Ds, diploid-simple

Concerning the clinical course, 8 out of 10 patients (80%) with aneuploid tumor had metastasis and 2 (20%) died of disease. Out of 27 patients with "simple-diploid" tumor, 8 patients (29.6%) had metastasis, 7 (25.9%) had recurrence, 4 (14.8%) died of disease and 8 (29.6%) are tumor-free. 4 of 9 patients (44.4%) with "complex-diploid" tumor had metastasis, 2 (22.2%) had recurrence, 1 (11.1%) died of disease and 2 (22.2%) had no recurrent tumor (Table 1). Using Fisher's exact test the three groups proved to be significantly different (P = 0.04) (Table 3).

**Table 3:** Use of Fisher's exact test whether the simple, complex diploid and aneuploid groups can be separated

Histogram	M	R	N	*Total*
Simple diploid	12	7	8	*27*
Complex diploid	5	2	2	*9*
Aneuploid	10	0	0	*10*
***Total***	*27*	*9*	*10*	*46*

The three-rows by three-columns contingency table should be used if the total size of the data set is no greater than N = 90 and/or any cell value is less than 5.

p = 0.040127

Abbreviations: M, metastasis; R, recurrence; N, neither recurrence nor metastasis

## Discussion

The treatment of SSs is still an unsolved issue. Although the specific chromosomal translocation characteristic to SSs and its outcome, the chimeric protein is well-known, appropriate targeted therapy could not be found. Its possible cause could be that the specific translocation is essential for the initiation of tumorgenesis, but in tumor progression (recurrence and metastasis) further genetic alterations also play a crucial role. Since these genetic alterations arise randomly (typical losses and gains of chromosomes or chromosomal segments are not known) [9,10], measurement of the total DNA content gains high importance in estimating the prognosis of the disease and accordingly, in the selection of the aggressiveness of the therapy. A current report of a biphasic SS case with genomic instability revealed great cytogenetic heterogeneity within chromosome numbers and the recurrent presence of dicentric chromosomes [17]. Nevertheless, whether and how the biological behavior of the tumor is affected by cytogenetic changes remains uncertain.

Some SSs recur or develop metastases after a long time, sometimes 5-10 years after the primary diagnosis while others are more aggressive. This can only be partly explained by "conventional" prognostic factors (patient's age, tumor size, extent of poorly differentiated areas and the resectability of the tumor). Karyotyping and CGH analyses provide excellent insight into the sum genetic alterations of the tumor, but they are considerably time consuming and expensive methods. Measurement of the sum DNA content by flow or image cytometry can be faster, easier and cheaper to perform. The aim of the study was to observe whether the frequently occurring diploid SSs could be divided into further subgroups which show correlation to simple or complex karyotype.

Although CGH is a screening method for DNA copy number changes of the entire genome, it only detects "average" genetic abnormalities of the tumor specimen. Furthermore, only aberrations involving losses or gains of DNA sequences can be detected, whereas balanced chromosome abnormalities, such as reciprocal translocations, inversions, and point mutations are not detectable [18]. CGH cannot recognize changes if the fraction of normal cells is high or if cells are polyploid. Therefore, normal findings may be false negative. The samples in our population contained high fraction (at least 85%) of tumor cells.

We observed in our samples the most frequent chromosome alterations described in SS: gain of chromosome 2, 8, 12p, 12q and loss in 3p14 [9,10]. The most likely candidate genes at 12q13-12q15 are *MDM2, CDK2, ERBB3, SAS *and *CDK4; *and *RASSF1 *at 3p21.3. A clear association was described between gain of *SAS *and poor prognosis [10].

Our results show a correlation between DNA-ploidy, fine-tuned DNA-ploidy and the HR-CGH results. Diploid complex cases show numerous genetic alterations whereas in diploid simple cases no detectable chromosome aberrations were found. The explanation of this phenomenon is not evident: the complex diploid tumors cannot be regarded as aneuploid, even if the complex karyotype shows evident aneuploidy (although at a more sensitive level). It may be explained as individual cells with 5c exceeding events (single cell aneuploidy) show that the case has complex karyotype usually developing some aneuploid cells. In contrary, in case of simple karyotype this phenomenon can not be detected. Little is known about the prognostic value of DNA ploidy (at flow and image cytometry level). El-Naggar *et al*. and Lopes *et al*. found that ploidy status (aneuploidy) correlated significantly with reduced patient survival in SS [19,20]. However, the vast majority of SSs are diploid and these diploid tumors are aggressive in several cases. Furthermore, our previous results did not find ploidy (diploid, aneuploid group) as an independent prognostic factor [8]. Conversely, promising results were published about the SS cases with complex karyotype that show an inverse correlation with prognosis.

We would like to find a feasible method to detect possible aberrations which may reflect the cytogenetic alterations within the diploid group. The detection of single cell aneuploidy in parallel to the diploid stemline was in correlation with the complex caryotype. Single cell aneuploidy is a well known phenomenon and its detection is applied e.g. in the diagnostics of cervical dysplasia-carcinoma. [21,22]. Although the 3 groups (simple diploid - complex diploid - aneuploid)were significantly different, we cannot be completely sure in the unequivocally good prognoses of the simple diploid cases (12 out of 27 cases developed metastasis), still the tendency is unambiguous. By detecting single cell aneuploidy in the diploid group, we know that it means a complex karyotype at cytogenetic level and so there is a simple, inexpensive and fast tool available to provide important complementary data.

There are numerous prognostic factors in case of SSs to consider and some of them are still a matter of debate e.g. type of translocation (*SSX1 *or *SSX2*) and the histology type (monophasic or biphasic) [2,4,7,9,11,20,23]. The clearest prognostic factor is the size of the tumor (above or below 5 cm) but unfortunately in most of the cases (except the childhood SSs), at the time of diagnosis the tumor is larger than 5 cm as has happened in all of our examined cases.

In summary, measurement of DNA ploidy and separation of the diploid group based on single cell aneuploidy might provide us useful complementary data which reflects well the cytogenetic results and accordingly it may help the oncologists to select the appropriate therapy.

## Conclusions

Although synovial sarcoma is the 3rd most commonly occurring mesenchymal tumor in young adults, usually with a highly aggressive clinical course, remarkable differences can be seen in the clinical outcome. We found a feasible method to detect possible aberrations which may reflect the cytogenetic alterations within the diploid group. The detection of single cell aneuploidy in parallel to the diploid stemline might provide us useful complementary data which reflects well the cytogenetic results and accordingly it may help the oncologists to select the appropriate therapy.

## Competing interests

The authors declare that they have no competing interests.

## Authors' contributions

ZSB participated in the design of the study and performed the Comperative Genomic Hybridisation. ZSSZ participated in the design of the study and performed the DNA measurements. MSZ participated in coordination of the study. IA participated in collecting clinical data. ZSP participated in collecting clinical data. LF helped to draft the manuscript and performed the statistical analysis. GP helped to perform the Comperative Genomic Hybridisation. YCH helped to draft the manuscript and helped to performe the DNA measurements. ZS conceived of the study, and participated in its design and coordination and helped to draft the manuscript. All authors read and approved the final manuscript.
